# Supplementary material for: The Effects on Immune Function and Digestive Health of Consuming the Skin and Flesh of Zespri® SunGold Kiwifruit (Actinidia Chinensis var. Chinensis ‘Zesy002’) in Healthy and IBS-Constipated Individuals
Source: Nutrients. 2020 May 18;12(5):1453. doi: 10.3390/nu12051453 (PMC7284715; doi:10.3390/nu12051453)
Supplement: Supplementary file 1 [file nutrients-12-01453-s001.zip › copy of daily diary.pdf]

ROME III ADULT QUESTIONNAIRE FOR CONSTIPATION

This questionnaire is to learn more about the health problems that some people have with constipation and help us understand the severity of the problem for each individual. It will take about ten minutes to complete. To answer each question, fill in the circle to the left of the correct answer. You may find that you have not had any of the symptoms that we will ask you about, when this happens you will be instructed to skip over the questions that do not apply to you. If you are not sure about an answer or you cannot remember the answer to a question, just answer the best you can. Please check that you have not left any questions out as you go.

THANK YOU

Q1: **In the last 3 months**, how often did you have discomfort or pain anywhere in your abdomen?

- Never (Please skip to Q9)
- Less than one day a month
- One day a month
- Two to three days a month
- One day a week
- More than one day a week
- Everyday

Q2: **For women**: Did this discomfort or pain occur only during your menstrual bleeding and not at other times?

- No
- Yes
- Does not apply because I have had the change in life (menopause)
- Does not apply because I am a male

Q3: Have you had this discomfort or pain 6 months or longer?

- No
- Yes

Q4: How often did this discomfort or pain get better or stop after you had a bowel movement?

- Never or rarely
- Sometimes
- Often
- Most of the time
- Always

Q5: When this discomfort or pain started, did you have more frequent bowel movements?

- Never or rarely
- Sometimes
- Often
- Most of the time
- Always

Q6: When this discomfort or pain started, did you have less frequent bowel movements?

- Never or rarely
- Sometimes
- Often
- Most of the time
- Always

Q7: When this discomfort or pain started, were your stools (bowel movements) looser?

- Never or rarely
- Sometimes
- Often
- Most of the time
- Always

Q8: When this discomfort or pain started, how often did you have harder stools?

- Never or rarely
- Sometimes
- Often
- Most of the time
- Always

Q9: **In the last 3 months**, how often did you have fewer than three bowel movements (0-2) a week?

- Never or rarely
- Sometimes
- Often
- Most of the time
- Always

Q10: **In the last 3 months**, how often did you have hard or lumpy stools?

- Never or rarely
- Sometimes
- Often
- Most of the time
- Always

Q11: **In the last 3 months**, how often did you strain during bowel movements?

- Never or rarely
- Sometimes
- Often
- Most of the time
- Always

Q12: **In the last 3 months**, how often did you have a feeling of incomplete emptying after bowel movements?

- Never or rarely
- Sometimes
- Often
- Most of the time
- Always

Q13: **In the last 3 months**, how often did you have a sensation that the stool could not be passed, (i.e., blocked), when having a bowel movement?

- Never or rarely
- Sometimes
- Often
- Most of the time
- Always

Q14: **In the last 3 months**, how often did you press on or around your bottom or remove stool in order to complete a bowel movement?

- Never or rarely
- Sometimes
- Often
- Most of the time
- Always

Q15: **In the last 3 months**, how often did you have difficulty relaxing or letting go to allow the stool to come out during a bowel movement?

- Never or rarely
- Sometimes
- Often
- Most of the time
- Always

Q16: Did any of the symptoms of constipation listed in questions 9-15 above begin more than 6 months ago?

- No
- Yes

Q17: **In the last 3 months**, how often did you have loose, mushy or watery stools?

- Never or rarely
- Sometimes
- Often
- Most of the time
- Always

A few further questions.....

Q18: **In the last three months**, how often have you noticed blood in your stools?

- Never or rarely
- Sometimes
- Often
- Most of the time
- Always

Q19: **In the last three months** how often have you noticed black stools?

- Never or rarely
- Sometimes
- Often
- Most of the time
- Always

Q20: have you been told by our doctor that you are anaemic (a low blood count or low iron)? (If female, not due to your menstrual period)

- No
- Yes

Q21; **In the last three months** have you unintentionally lost over 10 pounds (4.5 kg)?

- No
- Yes

Q22: If you are over the age of 50, have you had a recent major change in bowel movements (change in frequency or consistency)?

- No
- Yes
- Does not apply

Q23: Do you have a parent, brother or sister who has or has had one or more of the following?

- Cancer of the stomach, oesophagus or colon
- Ulcerative colitis or Crohn’s disease
- Coeliac disease
- Not applicable
