# Supplementary material for: The Effects on Immune Function and Digestive Health of Consuming the Skin and Flesh of Zespri® SunGold Kiwifruit (Actinidia Chinensis var. Chinensis ‘Zesy002’) in Healthy and IBS-Constipated Individuals
Source: Nutrients. 2020 May 18;12(5):1453. doi: 10.3390/nu12051453 (PMC7284715; doi:10.3390/nu12051453)
Supplement: Supplementary file 1 [file nutrients-12-01453-s001.zip › GSRS - US-English.pdf]

THE GASTROINTESTINAL SYMPTOM RATING SCALE (GSRS)

<u>Please read this first:</u>

This survey contains questions about how you have been feeling and what it has been like DURING THE PAST WEEK. Mark the choice that best applies to you and your situation with an “X” in the box.

1. Have you been bothered by PAIN OR DISCOMFORT IN YOUR UPPER ABDOMEN OR THE PIT OF YOUR STOMACH during the past week?

- ☐ No discomfort at all
- ☐ Minor discomfort
- ☐ Mild discomfort
- ☐ Moderate discomfort
- ☐ Moderately severe discomfort
- ☐ Severe discomfort
- ☐ Very severe discomfort

2. Have you been bothered by HEARTBURN during the past week? (By heartburn we mean an unpleasant stinging or burning sensation in the chest.)

- ☐ No discomfort at all
- ☐ Minor discomfort
- ☐ Mild discomfort
- ☐ Moderate discomfort
- ☐ Moderately severe discomfort
- ☐ Severe discomfort
- ☐ Very severe discomfort

©AstraZeneca, 1995. All rights reserved. (AMOS 97:04)
GSRS - US-English.

These questions should not be used, copied or distributed in any form without permission from AstraZeneca R&D, HEOR, SE-431 83 Mölndal, Sweden, PROinformation@astrazeneca.com

3. Have you been bothered by ACID REFLUX during the past week? (By acid reflux we mean the sensation of regurgitating small quantities of acid or flow of sour or bitter fluid from the stomach up to the throat.)

- ☐ No discomfort at all
- ☐ Minor discomfort
- ☐ Mild discomfort
- ☐ Moderate discomfort
- ☐ Moderately severe discomfort
- ☐ Severe discomfort
- ☐ Very severe discomfort

4. Have you been bothered by HUNGER PAINS in the stomach during the past week? (This hollow feeling in the stomach is associated with the need to eat between meals.)

- ☐ No discomfort at all
- ☐ Minor discomfort
- ☐ Mild discomfort
- ☐ Moderate discomfort
- ☐ Moderately severe discomfort
- ☐ Severe discomfort
- ☐ Very severe discomfort

5. Have you been bothered by NAUSEA during the past week? (By nausea we mean a feeling of wanting to throw up or vomit.)

- ☐ No discomfort at all
- ☐ Minor discomfort
- ☐ Mild discomfort
- ☐ Moderate discomfort
- ☐ Moderately severe discomfort
- ☐ Severe discomfort
- ☐ Very severe discomfort

©AstraZeneca, 1995. All rights reserved. (AMOS 97:04)
GSRS - US-English.

These questions should not be used, copied or distributed in any form without permission from AstraZeneca R&D, HEOR, SE-431 83 Mölndal, Sweden, PROinformation@astrazeneca.com

6. Have you been bothered by RUMBLING in your stomach during the past week? (Rumbling refers to vibrations or noise in the stomach.)

- ☐ No discomfort at all
- ☐ Minor discomfort
- ☐ Mild discomfort
- ☐ Moderate discomfort
- ☐ Moderately severe discomfort
- ☐ Severe discomfort
- ☐ Very severe discomfort

7. Has your stomach felt BLOATED during the past week? (Feeling bloated refers to swelling often associated with a sensation of gas or air in the stomach.)

- ☐ No discomfort at all
- ☐ Minor discomfort
- ☐ Mild discomfort
- ☐ Moderate discomfort
- ☐ Moderately severe discomfort
- ☐ Severe discomfort
- ☐ Very severe discomfort

8. Have you been bothered by BURPING during the past week? (Burping refers to bringing up air or gas from the stomach via the mouth, often associated with easing a bloated feeling.)

- ☐ No discomfort at all
- ☐ Minor discomfort
- ☐ Mild discomfort
- ☐ Moderate discomfort
- ☐ Moderately severe discomfort
- ☐ Severe discomfort
- ☐ Very severe discomfort

©AstraZeneca, 1995. All rights reserved. (AMOS 97:04)
GSRS - US-English.

These questions should not be used, copied or distributed in any form without permission from AstraZeneca R&D, HEOR, SE-431 83 Mölndal, Sweden, PROinformation@astrazeneca.com

9. Have you been bothered by PASSING GAS OR FLATUS during the past week? (Passing gas or flatus refers to the need to release air or gas from the bowel, often associated with easing a bloated feeling.)

- ☐ No discomfort at all
- ☐ Minor discomfort
- ☐ Mild discomfort
- ☐ Moderate discomfort
- ☐ Moderately severe discomfort
- ☐ Severe discomfort
- ☐ Very severe discomfort

10. Have you been bothered by CONSTIPATION during the past week? (Constipation refers to a reduced ability to empty the bowels.)

- ☐ No discomfort at all
- ☐ Minor discomfort
- ☐ Mild discomfort
- ☐ Moderate discomfort
- ☐ Moderately severe discomfort
- ☐ Severe discomfort
- ☐ Very severe discomfort

11. Have you been bothered by DIARRHEA during the past week? (Diarrhea refers to a too frequent emptying of the bowels.)

- ☐ No discomfort at all
- ☐ Minor discomfort
- ☐ Mild discomfort
- ☐ Moderate discomfort
- ☐ Moderately severe discomfort
- ☐ Severe discomfort
- ☐ Very severe discomfort

©AstraZeneca, 1995. All rights reserved. (AMOS 97:04)
GSRS - US-English.

These questions should not be used, copied or distributed in any form without permission from AstraZeneca R&D, HEOR, SE-431 83 Mölndal, Sweden, PROinformation@astrazeneca.com

12. Have you been bothered by LOOSE STOOLS during the past week? (If your stools (motions) have been alternately hard and loose, this question only refers to the extent you have been bothered by the stools being loose.)

- ☐ No discomfort at all
- ☐ Minor discomfort
- ☐ Mild discomfort
- ☐ Moderate discomfort
- ☐ Moderately severe discomfort
- ☐ Severe discomfort
- ☐ Very severe discomfort

13. Have you been bothered by HARD STOOLS during the past week? (If your stools (motions) have been alternately hard and loose, this question only refers to the extent you have been bothered by the stools being hard.)

- ☐ No discomfort at all
- ☐ Minor discomfort
- ☐ Mild discomfort
- ☐ Moderate discomfort
- ☐ Moderately severe discomfort
- ☐ Severe discomfort
- ☐ Very severe discomfort

14. Have you been bothered by an URGENT NEED TO HAVE A BOWEL MOVEMENT during the past week? (This urgent need to go to the toilet is often associated with a feeling that you are not in full control.)

- ☐ No discomfort at all
- ☐ Minor discomfort
- ☐ Mild discomfort
- ☐ Moderate discomfort
- ☐ Moderately severe discomfort
- ☐ Severe discomfort
- ☐ Very severe discomfort

©AstraZeneca, 1995. All rights reserved.

These questions should not be used, copied or distributed in any form without permission from AstraZeneca R&D, HEOR, SE-431 83 Mölndal, Sweden, PROinformation@astrazeneca.com

15. When going to the toilet during the past week, have you had the SENSATION OF NOT COMPLETELY EMPTYING THE BOWELS? (This feeling of incomplete emptying means that you still feel a need to pass more stool despite having exerted yourself to do so.)

- ☐ No discomfort at all
- ☐ Minor discomfort
- ☐ Mild discomfort
- ☐ Moderate discomfort
- ☐ Moderately severe discomfort
- ☐ Severe discomfort
- ☐ Very severe discomfort

PLEASE CHECK THAT ALL QUESTIONS HAVE BEEN ANSWERED!

THANK YOU FOR YOUR CO-OPERATION.

©AstraZeneca, 1995. All rights reserved. (AMOS 97:04)
GSRS - US-English.

These questions should not be used, copied or distributed in any form without permission from AstraZeneca R&D, HEOR, SE-431 83 Mölndal, Sweden, PROinformation@astrazeneca.com
