# Supplementary material for: The Effects on Immune Function and Digestive Health of Consuming the Skin and Flesh of Zespri® SunGold Kiwifruit (Actinidia Chinensis var. Chinensis ‘Zesy002’) in Healthy and IBS-Constipated Individuals
Source: Nutrients. 2020 May 18;12(5):1453. doi: 10.3390/nu12051453 (PMC7284715; doi:10.3390/nu12051453)
Supplement: Supplementary file 1 [file nutrients-12-01453-s001.zip › Participant Screening Questionnaire.pdf]

Participant Screening Questionnaire

Today's Date:

Please supply the following information:

Name: ____________________________________

Date of Birth: ____________________________________

Age: ____________________________________

General Practitioner (if known) ____________________________________

Please indicate the ethnicity that you ***primarily*** *identify with*:

NZ European □ Māori □ Samoan □ Cook Island Māori □ Tongan □ Niuean □ Chinese □ Indian □ Other (e.g. Malaysian, Japanese) □

Please state ___________________

Contact Details:

Home Address:	Work Address:
Telephone Number<br>Home:<br>Mobile:	Telephone Number<br>Home:<br>Mobile:
Email:	Email:
Which phone number/email would you prefer us to use to contact you?	
Can we leave a message with your partner/flatmates/family/Whanau or leave a message on your answer phone?<br>YES ☐ NO ☐	

If your contact details change, please inform us.

Emergency Contact Details:

In the unlikely event of an emergency, please list the name and contact details of the person you would like us to contact and their relationship to you.

We need to record your height and weight for the start of the study. Please ask the researcher present to make these measurements for you.

Height: ______________________________

Weight: _____________________________

BMI: ______________________________

Past Medical History

Please answer the following questions. If answering YES to any of the following, please give as many details as you can such as when you were diagnosed and if the condition is still ongoing.

HAVE YOU SUFFERED FROM	YES	NO	DETAILS
**Heart & Circulation**			
Heart attack or Angina			
Rheumatic Fever			
Heart murmurs			
High blood pressure			
Anaemia			
Other problems with your heart and circulation (please specify)			
**Lung**			
Asthma			
Bronchitis/Emphysema			
Tuberculosis (TB)			
Other lung problems (please specify)			

HAVE YOU SUFFERED FROM	YES	NO	DETAILS
**Digestive System**			
Stomach/duodenal ulcer			
Irritable Bowel Syndrome complicated by diarrhoea			
Irritable Bowel Syndrome complicated by constipation			
Crohn’s Disease			
Coeliac Disease			
Indigestion			
Constipation			
Other digestive problems (please specify)			
**Neurological System**			
Migraine (Number per year)			
Recurring headaches			
Fits			
Head injury with loss of consciousness			
Epilepsy			
Faints			
Blackouts			
Other neurological problems (please specify)			

HAVE YOU SUFFERED FROM	YES	NO	DETAILS
**Kidney/Liver/Serious Infections**			
Hepatitis			
Jaundice (at what age?)			
Kidney or gall stones			
Recurrent cystitis			
Other kidney/liver or serious infections (please specify)			
**Hormones**			
Diabetes			
Thyroid Disorder			
Other hormone problems (please specify)			
**Skin**			
Psoriasis			
Dermatitis			
Eczema			
Other skin problems (please specify)			
**Joint/Muscle/Bone**			
Arthritis			
Other (Please specify)			

HAVE YOU SUFFERED FROM	YES	NO	DETAILS
**Allergies**			
Hayfever			
Other allergies (please specify)<br>Exactly what are you allergic to?<br>What happens to you e.g. rash, swelling, breathing difficulties?			
Psychiatric			
Have you ever suffered or do you suffer with anxiety attacks or depression?<br><br>If YES, can you provide us with details of:<br>When this happened?<br>Was this caused by a specific event?<br>Your treatment e.g. did you require counselling, psychiatric referral, medication?			
Have you ever suffered or do you suffer with an eating disorder?			
Have you ever received treatment or a psychiatric disorder?			
**Hospital Attendance**			
Have you ever attended hospital<br>• As inpatient?<br>• As an outpatient?			
Have you ever undergone an operation or any surgical procedure? (Please indicate approximate dates)			

Please list any medications you are taking for your medical conditions. Please state the **dosage** and when you **started** taking this medication.

MEDICATION	Date Started	Dosage

Are you currently taking laxatives. If yes, what/dose/frequency?

____

____

Are you currently taking any dietary supplements? This includes products such as Metamucil and Benefibre? If yes, what/dose/frequency?

____

____

In the last two week have you consumed Yakult, Acitvate or any other probiotic fermented milk drink? If yes, which brand and approximately how much do you consume/week?

____

____

CURRENT HEALTH

Please answer the following questions. If answering YES to any of the following, please give as much detail as possible.

CURRENT HEALTH	YES	NO	DETAILS
**Smoking/Alcohol/Recreational Drugs**			
Do you smoke?<br>• If YES, how many per day?<br>• If a previous smoker, please state when you gave up<br>• How many did you smoke?<br>• Do you use nicotine replacement therapy?			
Do you drink alcohol?<br>• If YES, on average how many units a week do you drink?<br>(1 unit = ½ pint beer/ ~300ml or 1 glass of wine or 1 measured spirit)			
Do you take recreational drugs e.g. cannabis/ecstacy?			
**Immunisations/vaccinations**			
• Have you required travel or work related immunisations within the last 3 months?<br>• Are you undergoing a course of vaccinations?			
**Tattoos/Piercings**			
Do you have tattoos or body piercings?			

Physical Activity

Please indicate how much physical activity you participate in:

**LEVEL OF ACTIVITY**	Tick one
**Sedentary** – no exercise	
**Mild exercise –** i.e. climbing stairs, walk ~15 minutes, golf	
**Occasional vigorous exercise –** work or recreation, less than 4x/week for 30 minutes	
**Regular vigorous exercise –**work or recreation 4x/week for at least 30 minutes	

Other Information

Is there any other information, not discussed, that you feel relevant regarding your health?

____

____

FEMALE PARTICIPANTS ONLY

Are you post-menopausal? Yes/No

If not, can you please state the date of your last menstrual period? ______________________

How would you describe your menstrual cycle? Regular/Irregular

On average can you please state the number of days in your cycle? ______________________

Is it possible that you may be currently or may wish to become pregnant in the next 24 weeks? Yes/No

DECLARATION BY PARTICIPANT:

I (print name) ______________________________________ have given true and complete information to the best of my knowledge.

Signature: ____________________________________ Date: _____________________

Researcher: ___________________________________ Date: _____________________
